# P-1580. Impacts on Infectious Complications of Drug Use and Cost Effectiveness of a City-Wide Sales Tax to Address Mental Health and Substance Use Disorders

**DOI:** 10.1093/ofid/ofaf695.1759

**Published:** 2026-01-11

**Authors:** Kirk Fetters, Pranav Padmanabhan, Alia Al-Tayyib, Kristina Yamkovoy, Gwenyth Day, Danielle M Kline, Rebecca Ochtera, Lorez Meinhold, Joshua Barocas

**Affiliations:** University of Colorado School of Medicine, Division of Infectious Diseases, Denver, Colorado; University of Colorado School of Medicine, Division of General Internal Medicine, Denver, Colorado; Denver Health and Hospital Authority, Denver, Colorado; University of Colorado School of Medicine, Denver, Colorado; University of Colorado School of Medicine, Division of Pulmonary Sciences and Critical Care Medicine, Denver, Colorado; University of Colorado School of Medicine, Division of General Internal Medicine, Denver, Colorado; Caring for Denver Foundation, Denver, Colorado; Caring for Denver Foundation, Denver, Colorado; University of Colorado Anschutz Medical Campus, Aurora, CO

## Abstract

**Background:**

In 2018, Denver voters approved a 0.25% sales tax to fund mental health and substance misuse needs around the city. These funds are granted to organizations providing diverse services. Several grantees provide peer navigation services to improve linkage to medications for opioid use disorder (MOUD), which are effective at reducing opioid use, thereby possibly reducing risk for serious injection-related infections (SIRIs). The value of enhanced navigation services may only be realized over time given the relapsing nature of OUD. We assessed the impact of peer navigation services on SIRIs as well as the cost effectiveness.

**Methods:**

We used a closed cohort microsimulation model of the natural history of injection drug use to project the 10-year impact of the status quo (baseline access to services without supplemental funding for programs) to enhanced care (taxpayer-funded peer navigation services and MOUD integration). The model used city-level data from the CDC’s NHBS, published data, and grantee reports on improved linkage to MOUD. Costs were derived from literature, Centers for Medicaid and Medicare Services fee schedules, and the Medical Expenditure Panel Survey, using a modified societal perspective. Outcomes included cases of and subsequent hospitalizations for infective endocarditis (IE) and skin and soft tissue infections (SSTI), life years (LYs), costs, and incremental cost effectiveness ratios (ICERs).

**Results:**

Among the estimated 9,697 people who inject drugs in Denver, the status quo resulted in 1,240 cases of IE (880 hospitalizations), 14,190 severe SSTIs (12,570 hospitalizations), and 4.9 discounted LYs at a discounted cost of $272,770/person over a 10-year time horizon. Enhanced care led to 12.8% fewer cases of IE (11.4% decrease in IE-related hospitalizations), 4.2% decrease in SSTIs (8.6% increase in SSTI-related hospitalizations), a 1% increase in discounted LYs, and a 3.5% increase in discounted costs. The ICER for the enhanced care strategy was $206,000/discounted LY compared to status quo. The enhanced care strategy will cost each Denver taxpayer an additional $18/year.

**Conclusion:**

The peer navigation programs supported by Denver taxpayers that improve linkage to MOUD also improve infectious disease outcomes and life expectancy and may be cost effective.

**Disclosures:**

All Authors: No reported disclosures

